# Dispersal pathways and infection strategies within the Global Halophilic Virome

**DOI:** 10.1093/ismeco/ycag244

**Published:** 2026-08-25

**Authors:** Borja Aldeguer-Riquelme, Cristina López, Eva Mayol, María Dolores Ramos-Barbero, Valentin Gangloff, Tomeu Viver, Ana S Ramírez, Souad Amiour, Aharon Oren, Stephanus N Venter, María E Llames, Bernardo González, Horia L Banciu, Catherine Lizama, Ramon Rossello-Mora, Fernando Santos, Josefa Antón

**Affiliations:** Department of Physiology, Genetics, and Microbiology, University of Alicante, San Vicente del Raspeig 03690, Alicante, Spain; School of Civil & Environmental Engineering and School of Biological Sciences, Georgia Institute of Technology, Atlanta, GA 30332, United States; Department of Biomedicine and Dentistry, Faculty of Biomedical Sciences and Sports, Universidad Europea de Andalucía, Calle Eolo 10, 29010-Málaga, Spain; Department of Physiology, Genetics, and Microbiology, University of Alicante, San Vicente del Raspeig 03690, Alicante, Spain; Department of Physiology, Genetics, and Microbiology, University of Alicante, San Vicente del Raspeig 03690, Alicante, Spain; HUB Ambiental UPLA, Universidad de Playa Ancha, Valparaíso 2360004, Chile; Department of Physiology, Genetics, and Microbiology, University of Alicante, San Vicente del Raspeig 03690, Alicante, Spain; Departament de Genètica, Microbiologia i Estadística, Universitat de Barcelona, Diagonal 643. Annex. Floor 0, E-08028 Barcelona, Spain; Department of Physiology, Genetics, and Microbiology, University of Alicante, San Vicente del Raspeig 03690, Alicante, Spain; Marine Microbiology Group, Department of Animal and Microbial Biodiversity, Mediterranean Institute for Advanced Studies (IMEDEA, UIB-CSIC), Esporles 07190, Spain; Unidad de Epidemiología y Medicina Preventiva, IUSA, Facultad de Veterinaria, Universidad de Las Palmas de Gran Canaria, C/Trasmontaña s/n, Arucas 35413, Canary Islands, Spain; Laboratory of Bioengineering, National Higher School of Biotechnology, Ali Mendjeli University Town, E66 P.O. Box. Ali Mendjeli University Town, E66 P.O. Box. Constantine 25100, Algeria; The Institute of Life Sciences, The Hebrew University of Jerusalem, The Edmond J. Safra Campus, 9190401 Jerusalem, Israel; Department of Biochemistry, Genetics and Microbiology, and Forestry and Agricultural Biotechnology Institute (FABI), University of Pretoria, Hatfield, 0028, Pretoria, South Africa; Laboratorio de Ecología Microbiana Ambiental, Instituto Tecnológico de Chascomús (CONICET-UNSAM), Escuela de Bio y Nanotecnologías (UNSAM), B7130 Chascomús, Buenos Aires, Argentina; Facultad de Ingeniería y Ciencias, Universidad Adolfo Ibáñez–Center for Applied Ecology and Sustainability, Santiago de Chile 7941169, Chile; Department of Molecular Biology and Biotechnology, Faculty of Biology and Geology, Babeș-Bolyai University, 400006, Cluj-Napoca, Romania; Emil G. Racoviță Institute, Center for Astrobiology and Extreme Environments, Babeș-Bolyai University, 5-7 Clinicilor Street, 400006, Cluj-Napoca, Romania; Departamento de Tecnología Médica, Facultad de Ciencias de la Salud, Universidad de Antofagasta, Antofagasta 1240000, Chile; Marine Microbiology Group, Department of Animal and Microbial Biodiversity, Mediterranean Institute for Advanced Studies (IMEDEA, UIB-CSIC), Esporles 07190, Spain; Department of Physiology, Genetics, and Microbiology, University of Alicante, San Vicente del Raspeig 03690, Alicante, Spain; Applied Biomedicine Group (Alicante Institute for Health and Biomedical Research, ISABIAL), Alicante 03080, Spain; Department of Physiology, Genetics, and Microbiology, University of Alicante, San Vicente del Raspeig 03690, Alicante, Spain; Applied Biomedicine Group (Alicante Institute for Health and Biomedical Research, ISABIAL), Alicante 03080, Spain; Multidisciplinary Institute of Environmental Studies Ramon Margalef, Alicante 03690, Spain

**Keywords:** viral biogeography, viral dispersion, viral infection, hypersaline systems, haloviruses, extreme halophiles

## Abstract

Microbial biogeography has recently attracted significant attention for its potential to provide novel ecological and evolutionary insights, with environmental connectivity emerging as a key factor in the global distribution of viruses and their hosts. Hypersaline systems, due to their geographical isolation and extreme conditions, are typically expected to exhibit low connectivity. However, reports of similar viral assemblages in geographically distant hypersaline environments suggest a higher level of connectivity than previously assumed, although the mechanisms underlying viral dispersal remain poorly understood. We hypothesized that seawater facilitates the dispersal of halophilic viruses (haloviruses). To test the hypothesis, we analyzed twenty hypersaline water samples from globally distributed sites across four continents and constructed “The Global Halophilic Virome Dataset” (GHV), containing 10 705 viral Operational Taxonomic Units (vOTUs). Additionally, we proposed a sample-adapted threshold to distinguish between “abundant” and “rare” members of the virosphere. Our findings indicate that coastal hypersaline systems harbor highly similar viral communities, whereas inland systems are dominated by endemic vOTUs, and that seawater may act as the primary carrier of halovirus dispersal. Infection strategies also varied across environments, with coastal salterns showing a lower potential for viral infection and a higher percentage of viruses with a potential integrative temperate lifestyle.

## Introduction

Microbial community structure is mainly determined by processes of dispersion, selection, diversification, and drift [1]. In the case of viruses, dispersion is frequent in highly connected environments, such as seawater, due to the continuous movement and mixing of water. This is evidenced by the detection of the same viral genomes across different water masses worldwide [2]. In other environments, such as soils, viral dispersion is likely more limited, with studies reporting a high fraction of sample-specific viral genomes even among samples located <10 m apart [3]. Therefore, environmental connectivity is a key factor for viral dispersion. Indeed, the distribution of viruses at a global scale has gained the attention of the scientific community, as demonstrated by the large number of articles published during the last decade on the biogeography of viruses from a range of environments, including seawater [4–6], soils [7], the mammalian and human gut [8, 9], or activated sludge systems [10], among others. Understanding viral biogeography not only provides a framework to predict host interactions, but it also reveals how environmental and evolutionary pressures shape viral communities.

Within this framework, hypersaline environments, due to their geographical isolation and extreme conditions, should apparently have low connectivity with one another. However, several studies have reported the detection of closely related microbial and viral genomes in geographically distant hypersaline systems [11–15], suggesting that active dispersal pathways may be at play [16]. While some authors have proposed atmospheric dispersal as an important pathway in aquatic ecosystems [17, 18], others have instead pointed to the nostril glands and feathers of migratory birds as likely vectors for microbial dispersal among hypersaline systems [19–22]. Additionally, extreme halophiles have been cultured from seawater, suggesting that it could also act as a dissemination agent [23]. Nevertheless, the evidence supporting these three potential dispersal pathways (airborne transport, birds, or seawater) remains largely anecdotal, drawn primarily from studies of the cultivation of extreme halophiles, with no strong evidence confirming their migration. Recognizing the need for a comprehensive biogeographic study of globally distributed hypersaline systems to better understand the dispersal routes of extreme halophilic microorganisms, an international consortium launched the Halophile Sequencing Project (HSP) in 2018 [24], with the aim of expanding our understanding of the global distribution and composition of extreme halophilic prokaryotic communities. The sequencing of 25 brine metagenomes from coastal and inland sites across 11 countries revealed that prokaryotic communities from coastal locations were highly similar, suggesting an environmental connection potentially linked to unrestricted dispersal via ocean currents [25]. However, the viral fractions of these samples were not explored.

By infecting their hosts, viruses may play an important role in population control, horizontal gene transfer, and nutrient recycling [26]. The degree of these effects depends on the virus’s host range and infection strategy, which spans from strictly virulent (or purely lytic) to integrated temperate viruses, passing through chronic infection and pseudolysogeny [27]. However, the infection strategy employed by uncultured viruses in natural environments, and the factors that determine it, remain largely unknown. In this sense, hypersaline environments are again excellent systems for the study of viruses (halophilic viruses, or haloviruses) due to their high abundances (up to 10^9^ virus-like particles, or VLPs, per milliliter) and virus-to-cell ratios (>100 VLP/cell) [28], and their diverse infection strategies [12]. The relatively low microbial diversity in hypersaline environments compared to other more complex ecosystems such as seawater or marine sediments [28–30] simplifies the identification of viral-host pairs, enabling more precise viral-host associations than in more complex settings. Furthermore, the limited influence of bacterivory in most hypersaline environments close to salt saturation [28–30] may favor microbial population dynamics and evolution to be more directly attributed to viral activity.

In this work, we aim to unveil the distribution pathways and infection strategies of extremely halophilic viral assemblages at a global scale. Our working hypotheses are that haloviruses are effectively dispersed via seawater and that viral infection strategies could be influenced by the environment. To test these hypotheses, we analyzed the viromes and metagenomes of 20 water samples from different hypersaline systems distributed worldwide, collected over a short period (<16 months) as part of the HSP Project.

## Materials and methods

### Sampling and sample processing for viral DNA extraction

**Figure 1:**
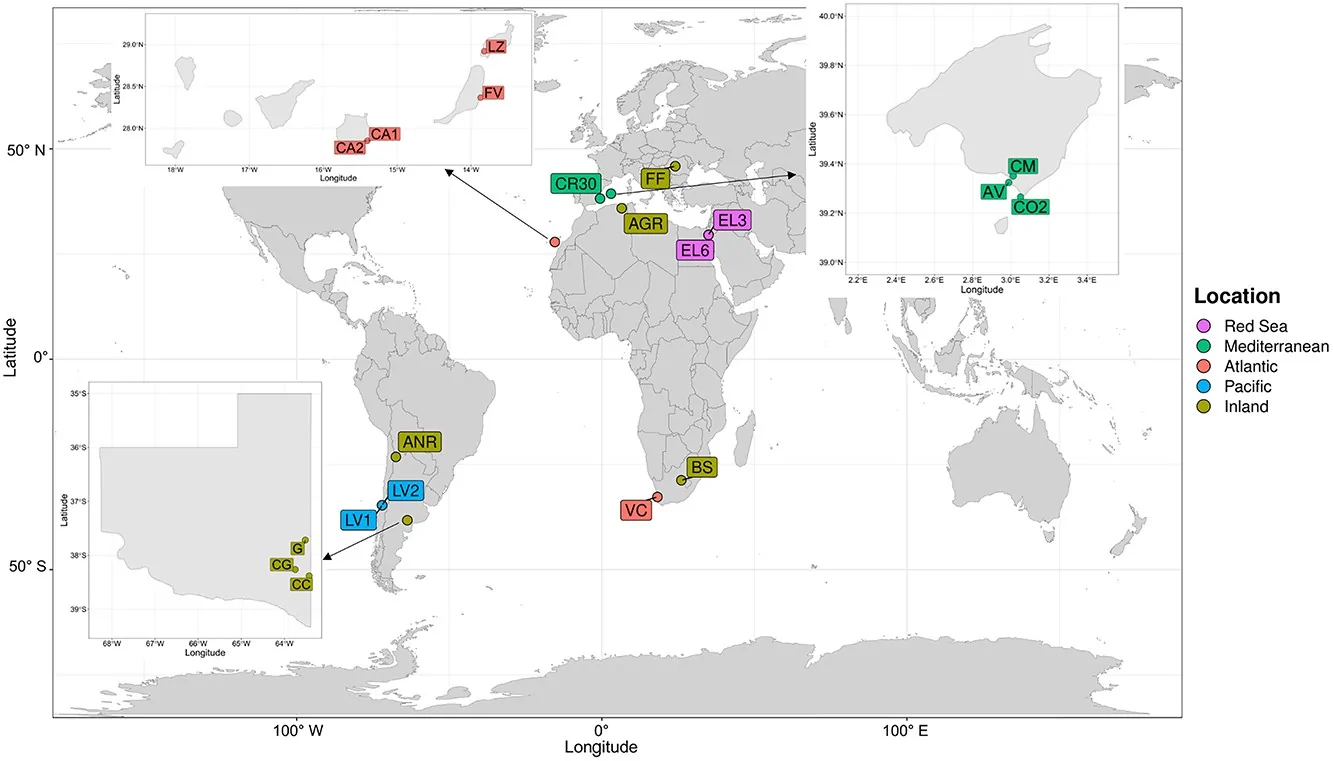
Global distribution of the 20 hypersaline samples analyzed in this study. Dots and labels are colored by their respective location (Red Sea: Violet; Mediterranean: Sea green; Atlantic: Salmon; Pacific: Sky Blue; and Inland: Olive green).

As described in the introduction, an international research consortium formed the HSP, comprising 19 researchers from 11 countries. For this study, the HSP team collected 20 water samples between September 2018 and January 2020 in 17 distinct hypersaline environments across seven countries in Asia, Europe, Africa, and America (Table 1, Fig. 1). The salinities of the samples were measured using a Sper Scientific Salt Refractometer (model number 300006; Arizona, USA) and the ionic compositions quantified by ion chromatography at the Technical Research Services of Alicante University (Spain), using a Metrohm, 850 ProfIC AnCat151-MCS system (Supplementary Table 1).

**Table 1:** Main features of the HSP samples used for the GHV dataset.

Country	Location		Abbreviation	Coordinates	Hypersaline system	Sampling date	Salinity (%)	Accession number	Gigabases
Spain	Mallorca	S’Avall	AV	39° 19′ 28.00″ N, 2° 59′ 21.00″ E	Coastal saltern (Mediterranean)	Sep 2018	36.2	ERR15142509	1.59
		Es Trenc	CM	39° 21′ 03.00″ N, 3° 00′ 45.00″ E	Coastal saltern (Mediterranean)	Sep 2018	34	ERR15142510	1.37
		Coco 2 - Mallorca	CO_2_	39° 15′ 55.00″ N, 3° 03′ 05.00″ E	Coastal pool (Mediterranean)	May 2019	30	ERR15142511	2.01
	Alicante	Santa Pola	CR30	38° 11′ 05.00″ N, 0° 35′ 08.00″ W	Coastal saltern (Mediterranean)	May 2019	37	ERR15142495	1.96
	Canary Islands	Janubio – Lanzarote	LZ	28° 55′ 11.00″ N, 13° 49′ 15.00″ W	Coastal saltern (Atlantic)	Jun 2019	34.4	ERR15142501	1.73
		Del Carmen - Fuerteventura	FV	28° 21′ 58.00″ N, 13° 52′ 12.00″ W	Coastal saltern (Atlantic)	Jul 2019	35	ERR15142500	1.71
		Arinaga 1 – Gran Canaria	CA1	27° 51′ 05.00″ N, 15° 24′ 07.00″ W	Coastal saltern (Atlantic)	Oct 2018	36	ERR15142498	1.54
		Arinaga 2 – Gran Canaria	CA2	27° 51′ 05.00″ N, 15° 24′ 07.00″ W	Coastal saltern (Atlantic)	Oct 2018	34	ERR15142499	1.91
Algeria	Oum El Bouaghi	Sebkha Ezzemoul	AGR	35° 53′ 13.90″ N, 6° 30′ 50.40″ E	Inland lake	Jan 2020	30	ERR15142494	1.72
Israel	Eilat	Eilat 3	EL3	29° 33′ 29.00″ N, 34° 57′ 54.00″ E	Coastal saltern (Red Sea)	Sep 2018	33	ERR15142502	1.50
		Eilat 6	EL6	29° 33′ 29.00″ N, 34° 57′ 54.00″ E	Coastal saltern (Red Sea)	Sep 2018	34	ERR15142503	1.09
Romania	Transylvania	Fără Fund	FF	45° 52′ 34.00″ N, 24° 04′ 03.00″ E	Inland lake	Apr 2019	29	ERR15142512	1.74
Chile	Boyeruca	Lo Valdivia 1	LV1	34° 41′ 56.00″ S, 72° 00′ 44.00″ W	Coastal saltern (Pacific)	Jan 2019	26	ERR15142507	1.58
		Lo Valdivia 2	LV2	34° 41′ 56.00″ S, 72° 00′ 44.00″ W	Coastal saltern (Pacific)	Jan 2019	26	ERR15142508	1.38
	Antofagasta	Pujsa	ANR	23° 12′ 20.00″ S, 67° 29′ 49.00″ W	Inland saltern	Sep 2018	15	ERR15142496	1.11
Argentina	La Pampa	Guatraché	G	37° 42′ 59.00″ S, 63° 31′ 04.00″ W	Inland lake	Jul 2019	33	ERR15142506	1.50
		Colorada Chica	CC	38° 22′ 51.00″ S, 63° 25′ 41.00″ W	Inland lake	Aug 2019	33	ERR15142504	1.65
		Colorada Grande	CG	38° 15′ 41.00″ S, 63° 45′ 02.00″ W	Inland lake	Aug 2019	32	ERR15142505	1.98
South Africa	Western Cape	Velddrif	VC	32° 44′ 03.00″ S, 18° 12′ 00.00″ E	Coastal saltern (Atlantic)	Jan 2019	34	ERR15142513	0.99
	Bloemfontein	Soutpan	BS	28° 45′ 21.00″ S, 26° 02′ 55.00″ E	Inland saltern	Jan 2019	25	ERR15142497	1.08

For virus recovery, around 1 liter of each brine was filtered through 0.2 μm Durapore filters (Merck, Darmstadt, Germany). The filtered volumes, containing the viruses, were then concentrated by tangential flow filtration (through a Vivaflow filter cassette system with a molecular weight cutoff of 30 000 Daltons; Sartorius, Göttingen, Germany). Then, 10 μl of every sample, equivalent to 0.5 ml of original sample, were fixed with formaldehyde (final concentration, 4%) and filtered through 0.2 μm to detect the presence of contaminating cells by 4′,6-diamidino-2-phenylindole (DAPI) staining [31] prior to ultracentrifugation at 286 000 *g* (Beckman Coulter centrifuge, rotor SW41TI) for 4 h at 20°C (whenever cells were detected, the concentrates were filtered again through 0.2 μm and the DAPI staining was repeated). Viral pellets were resuspended with ~400 μl of sterile artificial 25% sea water, mixed with equal volumes of 1.6% low-melting-point agarose (Condalab; Torrejón de Ardoz, Madrid, Spain), dispensed into 100 μl molds, and allowed to solidify at 4°C. Once solidified, agarose plugs were treated with two units of DNase I (SIGMA) for 1.5 h at 37°C to eliminate free DNA. Plugs were then immersed in ESP (0.5 M EDTA, pH 9.0; 1% N-laurylsarcosine, 1 mg/ml proteinase K), at a rate of 1 ml of ESP for every three plugs, and incubated at 50°C for 16 h to inactivate DNase and digest viral capsids. Prior to viral DNA extraction, ESP was removed by washing the plugs with Pefabloc® TE (10 mM Tris–HCL, pH 8; 1 mM EDTA, pH 8) (1.5 mM, Invitrogen; Waltham, Massachusetts, U.S.), for 4 h at 37°C, six subsequent washes with TE buffer (10 Mm Tris–HCl, 1 mM EDTA) at room temperature (40 minutes per wash), and a final wash low EDTA TE buffer (0.45 Mm Tris–HCl, 0.1 mM EDTA). The TE buffer was removed, plugs were melted for 10 min at 70°C, tempered at 42°C, and the agarose was digested with 25 units of β-agarase (New England Biolabs) per 100 mg of agarose for 2 h at 42°C. Samples were centrifuged for 10 min at 17 000 *g* to remove undigested agarose and viral DNA, then washed and concentrated using Amicon® Ultra-100 K devices (Merck, Darmstadt, Germany) with a retention volume of 20 μl. Viral DNAs were recovered in sterile ultrapure water and their concentrations determined by Qubit®, using the high-sensitivity kit. To assess cellular DNA contamination, extracted viral DNA samples were analyzed by Polymerase Chain Reaction (PCR) using universal 16S rRNA gene Illumina primers as in [31]. An internal positive control DNA was included in each viral DNA sample to rule out false-negative PCR results caused by PCR inhibitors. All reactions yielded negative results.

### Sequencing, assembly, and vOTUs identification

Metagenomic DNAs from extracellular viromes were sequenced at FISABIO (Valencia, Spain), using a MiSeq 2 × 300 bp run (Illumina) with Nextera XT protocol. Bioinformatic tools were used with default parameters unless otherwise indicated. Raw reads were processed with Trimmomatic-0.36 [32] to remove adapters, reads shorter than 36 bp, and low-quality bases (phred quality below 15 in a 4-base-wide sliding window). Then, FastQC v0.11.9 [33] was used to evaluate the quality of trimmed reads, and viromeQC v1.0 [34] was used to quantify viral sequence enrichment and the percentage of rRNA gene sequences. In addition, viromes’ diversity coverages were assessed using Nonpareil v3.4.0 [35] with the following settings: -T kmer -f fastq -X 50000. To compare viromes, distances among reads were calculated using MASH [36] and visualized as a Non-metric MultiDimensional scaling (NMDS) plot in R with the ggplot2 package [37]. Virome assemblies were performed independently for each sample using metaSPAdes v3.15.4 (k = 11, 33, 55, 77, 127) [38], Megahit v1.2.9 [39], and IDBA v1.1.3 (−-pre_correction) [40]. To identify species-rank virus groups (hereinafter vOTUs, from viral Operational Taxonomic Units), all contigs larger than 5 kb from the three assemblers were selected and clustered with cd-hit-est v4.8.1 [41] at 95% identity and 85% coverage, following previously recommended thresholds [42], yielding 10 505 vOTUs. In addition, 200 publicly available haloviral genomes from the National Center for Biotechnology (NCBI) database and recent publications [43, 44] were included to construct the “Global Halophilic Virome” (GHV) database, comprising 10 705 haloviral sequences. Software geNomad v1.8.1 [45] was used for the taxonomic assignment of vOTUs.

### Ubiquity, abundance, and microdiversity analyses

To determine the geographic distribution of each vOTU in our data set, the trimmed reads from each of the aforementioned 20 samples were aligned against the GHV using BLASTn [46]. Hits were filtered by best hit (if one read presented exactly the same hit against two vOTUs, both hits were kept), 95% minimum identity, and 70% coverage. A vOTU was considered present in a given sample if at least 85% of its genome length was covered by reads. UpSet plots [47], based on presence/absence data, were drawn to visualize the distribution of the vOTUs. Using the same data, interaction networks were constructed with ggraph (https://github.com/thomasp85/ggraph) to evaluate the connections among samples, that is, their percentages of shared vOTUs. The relative abundance of each vOTU in every sample was calculated as the number of recruited reads/genome size (bp)/virome size (number of total reads), and plots were drawn with ggplot2. Microdiversity calculations, based on the average nucleotide identity (>95%) of mapped reads (ANIr) [48, 49], were derived from BLASTn recruitments, considering only vOTUs with a minimum sequencing depth of 10.

### Host predictions

Prior to the identification of virus-host pairs, the 16S rRNA gene reads from the 20 cellular metagenomes corresponding to each one of the 20 viromes were identified and extracted with SortMeRNA v4.3.6 using the default database [50] and then taxonomically classified against the SILVA r138 database using BLASTn (95% minimum identity and 70% coverage). Based on this information, 5824 genomes of the kingdom *Nanobdellati* (2009), the class *Halobacteria* (3267), and the genera *Salinibacter* (169), *Spiribacter* (18), *Aquisalimonas* (9), *Dactylococcopsis* (1), *Psychroflexus* (26), *Owenweeksia* (8), *Salipaludibacillus* (11), *Salibaculum* (2), *Roseovarius* (102), *Roseibaca* (6), and *Paracoccus* (196) were downloaded from the NCBI genome database. In addition to publicly available genomes, the 528 metagenome-assembled genomes (MAGs) recovered from the cellular metagenomes [25] were also included. Virus-host pairs were then predicted by searching for CRISPR protospacers, tRNA homologies, and BLASTn hits of vOTUs against cellular genomes (to ensure the reliability of our results, only the CRISPR approach was used for MAGs recovered from cellular metagenomes). CRISPR spacers were identified from the cellular genomes and MAGs using the CRISPRCasFinder tool v4.2.20, retaining only those with evidence levels 3 or 4 (strongest evidence). The spacers were then queried against the vOTUs using BLASTn (−task blastn-short), and hits with>95% identity and >90% coverage were retained. tRNAScan-SE v2.0.9 [51] was employed to detect tRNAs in both cellular genomes and vOTUs. Transfer RNA sequences were then BLASTn-queried, retaining hits with >90% identity and >90% coverage [52]. Finally, vOTUs were BLASTn-queried against the cellular genomes to identify integrated sequences, considering as positive hits >1 kb in length and >95% identity [52].

### vOTUs annotation

Open Reading Frames (ORFs) were predicted for each vOTU using VirClust v2.0 [53]. Amino acid sequences were then annotated using Interproscan 5.57–90.0 [54], with the Pfam database [55], and VirClust v2.0 [53], with the pVOGs, VOGDB, PHROGS, and Efam databases [56–59].

### Statistical analyses

To perform multivariate statistical tests, the assumption of homogeneity of variances was first checked using betadisper, and then Analysis of Variance (ANOVA) was applied to test whether variances among groups differed significantly. In cases where homogeneity of variance was confirmed, PERMANOVA tests were carried out using the adonis2 function with 4999 permutations and Bray-Curtis distances. All functions used in this test are from the vegan package v2.6.4 [60].

## Results and discussion

### The GHV

**Figure 2:**
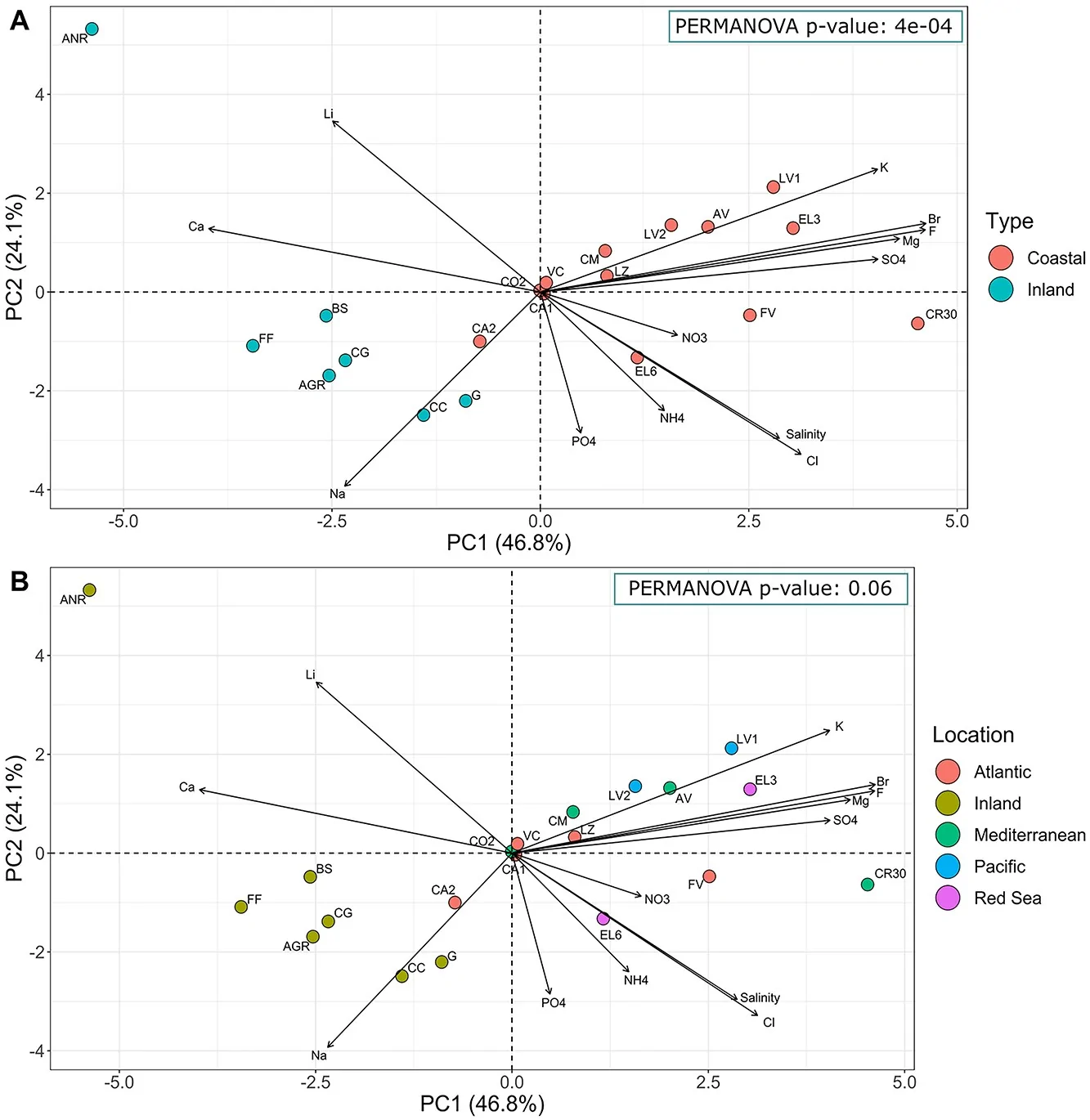
Principal component analysis (PCA) of the ionic composition of 20 hypersaline water samples. Samples are colored by sample type in A (Coastal: red, Inland: cyan); A) and geographic location in B (Red Sea: Violet; Mediterranean: Sea green; Atlantic: Salmon; Pacific: Sky Blue; and Inland: Olive green) (B) A significant clustering pattern based on sample type is observed, highlighting distinct ionic profiles between coastal and inland hypersaline waters.

The coordinated work of the HSP partners enabled sampling of 20 hypersaline thalassohaline waters from four continents (Africa, America, Asia, and Europe), with salinities ranging from 15% to 37% (Fig. 1, Table 1). Brines were collected over a short time scale (~1 year) and processed using the same protocol to obtain subsequent viromes. As in Viver *et al.* [25], a preliminary analysis based on the ionic composition clustered the hypersaline water samples in coastal (including Mediterranean, Red Sea, Atlantic, and Pacific locations) and inland (PERMANOVA *P*-value: .004; Fig. 2A). Although no statistically significant differences were observed among locations within each group (PERMANOVA *P*-value: .06; Fig. 2B), inland and coastal samples appeared clearly separated.

Good quality viromes were obtained for all HSP samples, as shown by high enrichments in viral reads (viromeQC scores >6.4×; threshold 3×) [34], low abundance of 16S rRNA gene sequences (<0.003%; threshold 0.02%) [61], and high nonpareil coverage values (>87%; threshold: >60%) [62] (Supplementary Table 2). When the trimmed reads of the HSP viromes were compared with a selection of soil, marine, and freshwater viral metagenomes, halophilic viral assemblages formed a cohesive group based on the MASH distances analysis (PERMANOVA *P*-value: 2e^−4^; Supplementary Fig. 1A). This result reinforces the existence of a “hypersaline-ness”, that is, a characteristic DNA signature of haloviruses. This concept, which was suggested >15 years ago with a reduced number of available viromes [28], has finally been well settled in this work with larger datasets. The same MASH analysis, only using the HSP virome reads, produced a cluster comprising most coastal sites (PERMANOVA *P*-value: .0172), strongly influenced by their geographical locations (Supplementary Fig. 1B). Viromes from inland hypersaline environments, instead, appeared more heterogeneous in their genetic composition, as indicated by higher dispersion in the plot (betadisper: average dispersion-to-median was 0.0974 and 0.1438 for coastal and inland, respectively). Similarly, total metagenomes from coastal sites showed higher genetic similarity among them than those from inland sites [25].

A total of 18 427 contigs longer than 5 kb were obtained from the independent assembly of each HSP virome. Given that it has been widely reported that analysis of the assembled fraction of a given metagenome may not be representative of its total reads [44, 63, 64], three approaches were employed to ensure the representativeness of the haloviral genome dataset. Considering the reported high microdiversity in halophilic viruses [28], we first leveraged different assembly tools (see the methods section) to minimize the biases that microdiversity imposes on the assembly process [2]. Second, we combined all HSP viral contigs above 5 kb into a single database, and the genomes of 213 publicly available cultured and uncultured haloviruses were added to the dataset. Then, vOTUs were constructed as defined previously [42], yielding a total of 10705 non-redundant sequences, whose representative sequences may correspond to complete viral genomes or genomic fragments (Supplementary Table 3). Of these, 7962 (74.4%) were classified as viral by geNomad [45]. Manual inspection of the functional annotation (Supplementary Table 4) of the remaining unclassified vOTUs identified an additional 782 sequences encoding viral hallmark genes, including integrases, portal proteins, tail proteins, capsid proteins, and terminases, among others. Consequently, the proportion of confidently identified viral sequences in our dataset increased to 82% (mean across samples: 81% ± 6.6%; range: 64%–98%). For the remaining 18% (1961 vOTUs), functional annotation did not reveal any clear cellular signature. Instead, these sequences encoded genes commonly found in both viruses and cellular organisms (e.g. DNA methyltransferases, nucleases, peptidases, and phosphoadenosine phosphosulfate (PAPS) reductases, all frequently reported in halophilic viruses), which were typically flanked by hypothetical proteins, consistent with a viral genomic context. Furthermore, 64% of these unclassified vOTUs were detected in a single sample, and 93% belonged to the rare virosphere (see below), indicating that their influence on the downstream analyses was negligible.

Taxonomically, the vast majority of vOTUs (75%) were assigned to the class *Caudoviricetes*, which comprises prokaryotic tailed dsDNA viruses. Approximately 1% were classified as pleomorphic archaeal viruses within the class *Huolimaviricetes* (family *Pleolipoviridae*), followed by ~0.3% corresponding to lemon-shaped viruses (family *Bicaudaviridae*) and same proportion of tailless icosahedral dsDNA phages from class *Laserviricetes*. All remaining viral groups accounted for <0.3% of the identified vOTUs. Notably, among the 99 vOTUs whose representative sequence corresponded to an isolated virus (or an environmental sequence with an established taxonomy), taxonomic assignment was accurate to the class level for dsDNA prokaryotic viruses (*Caudoviricetes* or *Laserviricetes*) and to the family level for pleomorphic viruses.

Putative hosts were inferred for 1029 vOTUs (9.6%) through CRISPR spacer matches, tRNA similarity, and BLASTn sequence identity (Supplementary Table 3). These vOTUs were tentatively associated with unclassified *Halobacteria* (286), *Halorubrum* (255), *Salinibacter* (215), *Haloquadratum* (63), and *Nanohaloarchaeota* (43). Among the same set of 99 vOTUs with experimentally confirmed hosts, host prediction was correct for 58% (34 vOTUs at the genus level and for an additional 23 at the class level). Only two vOTUs (vOTU_05952 and vOTU_08293), both classified by geNomad as members of the phylum *Nucleocytoviricota* (eukaryotic viruses), were incorrectly linked to *Nanohaloarchaeota* and *Salinibacter*, respectively.

In accordance, *Halorubrum*, *Salinibacter*, and *Haloquadratum* were the most ubiquitous hosts among the MAGs in the corresponding coastal and inland metagenomes [25]. Notably, inland halophilic viromes exhibited lower percentages of virus–host associations, most likely due to their higher diversity compared to coastal sites and the extensive “microbial dark matter” that characterizes these systems [25]. This represents, to our knowledge, the largest collection of halophilic viral sequences currently available, hereafter referred to as “The Global Halophilic Virome dataset”.

### Coastal and inland hypersaline environments harbor distinctive, abundant viral assemblages

**Figure 3:**
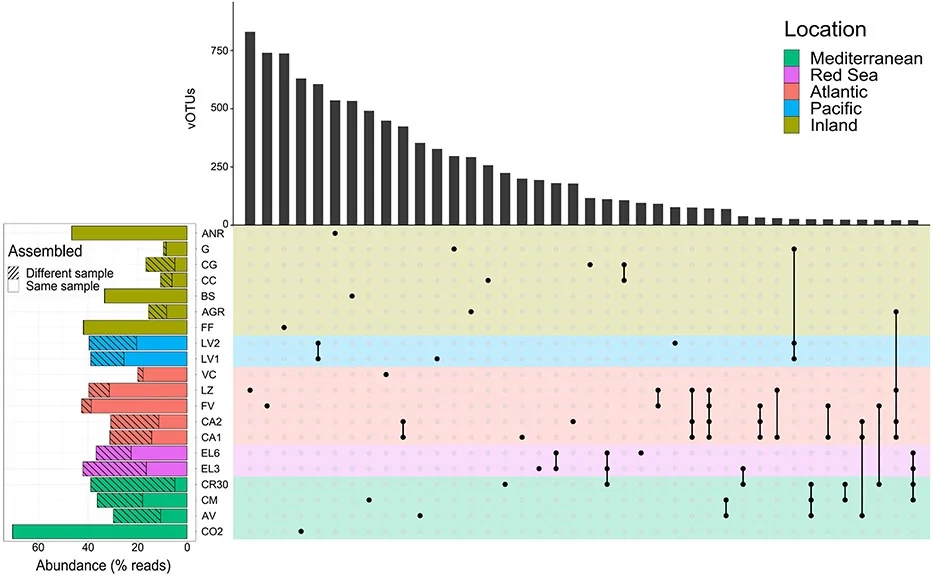
Distribution of the vOTUs in the GHV across the 20 hypersaline viromes. Bars at the top indicate the number of vOTUs displaying the distribution pattern shown in the panel below. For example, the first bar indicates there are approximately 800 vOTUs specific of sample LZ. Note that site-specific vOTUs are the most frequent, followed by vOTUs shared among geographically close locations. Panel on the bottom left displays a bar plot that shows, for each sample, the percentage of reads mapped by vOTUs recovered from the same (solid pattern) or a different sample (diagonal lines).

The presence or absence of vOTUs was used to define biogeographic patterns within the GHV at the time of sampling. Site-specific vOTUs were the most frequent and accounted for 70.6% of the GHV (Fig. 3), underscoring the relevance of endemic viral species, especially in inland environments (coastal samples: 40.2% ± 27.3%; inland samples: 72.8% ± 27.6%; *t-*test *P*-value .025). This result, which contrasts with the percentage of MAGs that were found to be locally restricted (26%; [25]), may be consistent with high viral specialization and local adaptation, likely driven by narrow host ranges and the rapid evolutionary dynamics and genomic plasticity characteristic of viral populations [65]. Although less frequent, we identified 2968 cosmopolitan vOTUs (defined here as those present in ≥2 samples), primarily in geographically proximate coastal environments. Only 1 vOTU was detected in 12 samples, whereas 22 vOTUs were detected in >10 samples, including all coastal sites plus the inland systems AGR (in Algeria) and CC/CG (in Argentina). In addition, recruitment against the GHV indicated that the set of vOTUs accounted for 9.5%–70.5% of each HSP virome reads (mean 33.5%) (Fig. 3, left). Remarkably, some viromes, such as those from CR30 (Alicante, Spain) and AV (Mallorca, Spain), showed a higher percentage of recruitment to vOTUs assembled from other samples than to their own vOTUs. This observation is likely related to the significantly higher viral microdiversity (ANIr) of the vOTUs assembled in other samples compared to those assembled in CR30 and AV (Supplementary Fig. 2), which probably affected their assemblage as previously demonstrated [2]. Therefore, our strategy enabled the detection of viral sequences in a virome from which they were not assembled, and our results supported the use of GHV as a good representative of HSP viromes. An NMDS plot, based on vOTU diversity and relative abundances, showed a sample distribution very similar to that obtained from the non-assembled reads (Supplementary Fig. 3).

**Figure 4:**
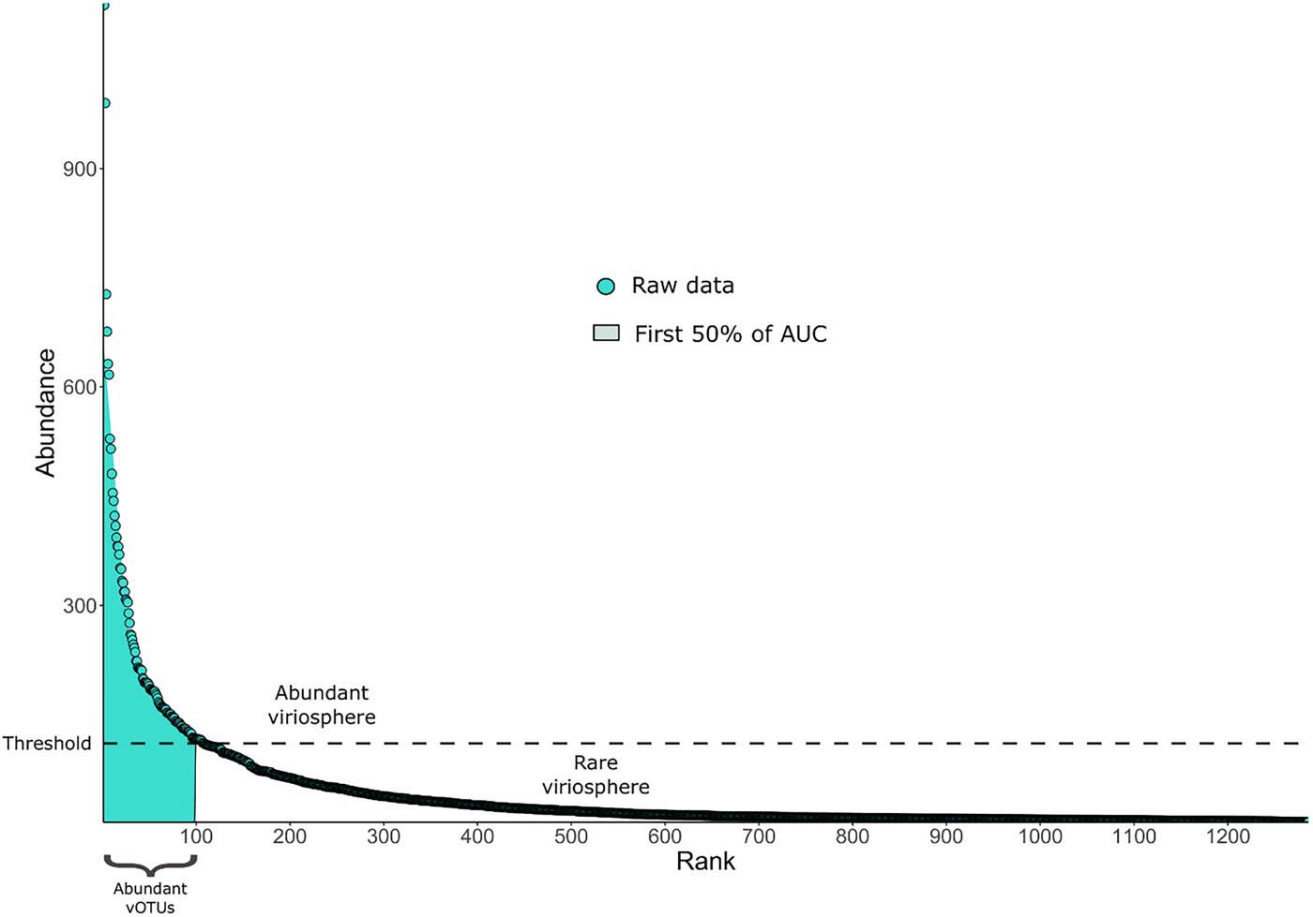
Taxon-rank plot illustrating the determination of a sample-adaptive threshold for defining abundant viral species. vOTUs are ranked along the x-axis by decreasing abundance (y-axis). The sample-adaptive threshold corresponds to the set of vOTUs accounting for the first 50% of the total AUC, represented by the shaded gray area. The horizontal dashed line marks the abundance cutoff separating the abundant from the rare virosphere.

Because a vOTU was categorized as present in a given virome when ≥85% of its genome was covered by reads (see the methods section), the presence/absence determinations may be biased against low-abundant viruses, which, although present in a given sample, would be difficult to detect depending on the sequencing effort. Thus, we decided to focus on the biogeographical distribution of the abundant vOTUs within the GHV. However, the definition of an “abundant” microbial species in microbial ecology is often vague, and the literature provides few clues on how to determine it throughout its extent. For cells, a threshold of 1% of the total community was proposed two decades ago [66]. For viruses, some authors have classified abundant viruses just by quantitative comparison with other viruses [2, 67]. However, quantitative comparisons do not necessarily allow sorting viral species (or vOTUs) into abundant or rare categories, and fixed thresholds are sometimes arbitrary and ignore the impact of diversity within a given sample. To circumvent this obstacle, we propose using a sample-adapted threshold in the field of Viral Ecology. This adaptive threshold is based on the taxon rank curve of a given sample, so it considers not only the relative abundance of a vOTU but also the sample’s viral diversity. By using this threshold, we have defined as abundant vOTUs those representing the first half (50%) of the total area under the curve (AUC) in the taxon rank plot, which is independent on the curve shape and the community structure (Fig. 4). It is important to note that a significant fraction of a given virome may consist of unassembled reads. In this sense, a viral sequence that does not assemble cannot belong to an abundant viral species (or vOTU).

**Figure 5:**
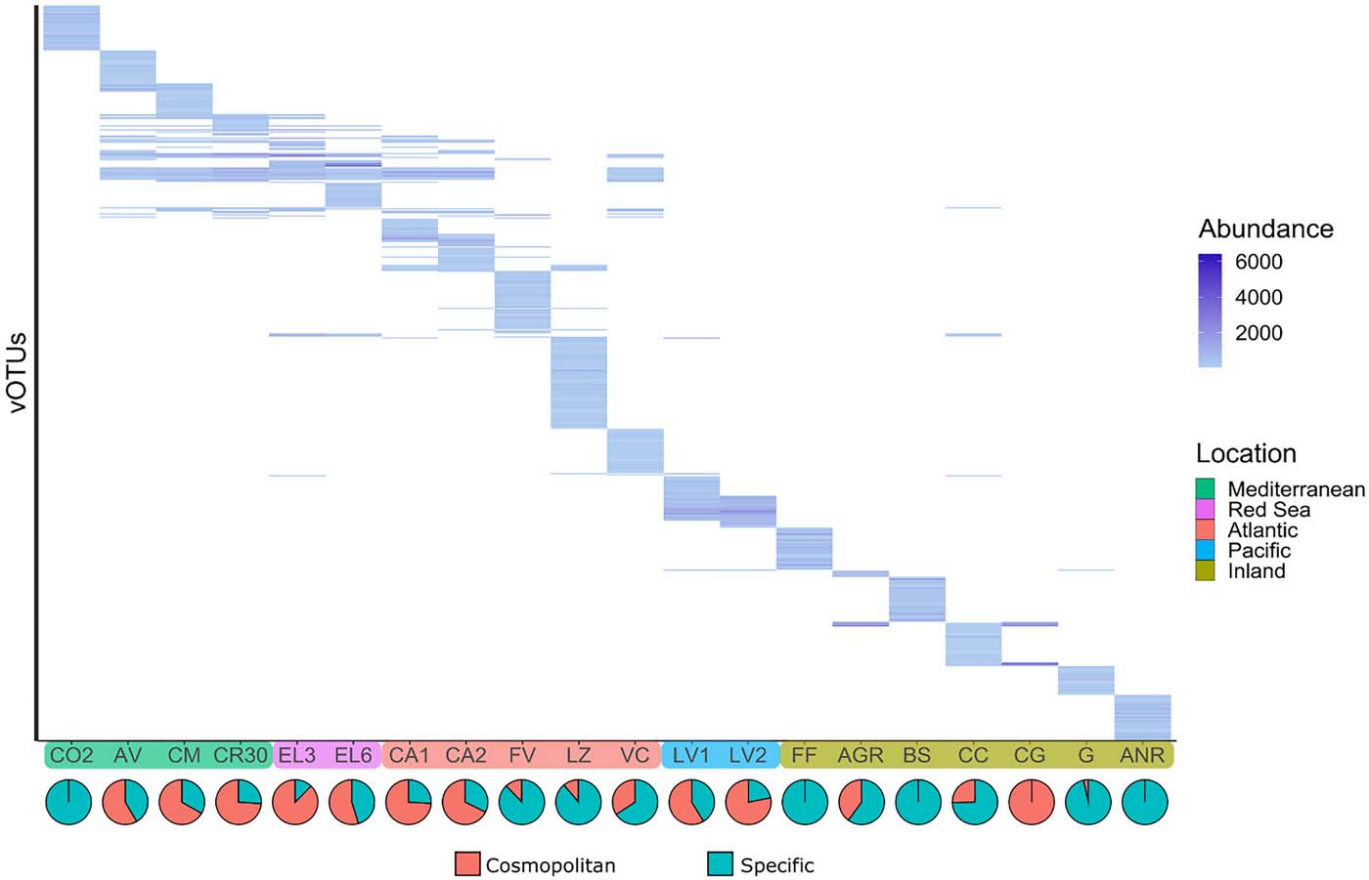
Distribution of the abundant vOTUs across the 20 sampled hypersaline environments. The heatmap displays the relative abundance of vOTUs (y-axis) for each sampling site (x-axis), represented by a blue gradient (darker shades indicate higher abundance). Colored background of x-axis labels indicates the sampling location (Mediterranean: Sea green; Red Sea: Violet; Atlantic: Salmon; Pacific: Sky Blue; and Inland: Olive green) following the same color scheme as in Figs 1 and 2. Pie charts below the heatmap illustrate the proportions of cosmopolitan (red, detected in >1 sample) and sample-specific vOTUs (blue, detected in 1 sample) found in each sample. Note that coastal hypersaline environments (left) are dominated by cosmopolitan vOTUs, whereas inland systems (right) harbor mainly sample-specific vOTUs.

By applying a sample-adapted threshold to our GHV, we identified 717 abundant vOTUs. Each of them recruited >0.1% of reads in the viromes where they were detected. We observed that abundant vOTUs were commonly shared among coastal and geographically close sampling sites, while those abundant in inland salterns were primarily site-specific (Fig. 5). Their putative hosts were mostly unknown. However, some abundant vOTUs were predicted to infect *Haloquadratum* (3.30%), *Salinibacter* (3.06%), and *Halorubrum* species (2.65%).

### A proposed model for halovirus dispersion

Early reports identified haloviral sequences from a coastal saltern in Spain that were also detected in a saltern pond in California [68]. In our study, the fact that abundant vOTUs were primarily shared among coastal environments (Fig. 5) reinforces connectivity among these sites, in contrast to inland environments. Also, some studies have reported the presence of ubiquitous microbial species in coastal salterns, but not in their inland counterparts [69]. For example, as discussed in [70], metagenomic sequences from Lake Tyrrell, an inland hypersaline lake in Australia, showed very low similarity to other globally distributed hypersaline samples [71]. More recently, Viver and co-workers [25] have found that 62.5% of the 284 genomospecies recovered from HSP cellular metagenomes were present across multiple inland or coastal hypersaline systems. However, taxonomic and genetic similarities were notably higher among species from coastal environments, highlighting their greater degree of environmental connectivity, possibly driven by ocean current dynamics. Taken together, these findings led us to hypothesize that marine currents would also serve as the primary mechanism for dispersing halophilic viruses among coastal hypersaline systems, based on three lines of evidence.

First, the observed similarity among coastal samples, in contrast to the pronounced dissimilarity among inland samples, can be explained by dispersion via seawater. Alternative pathways, such as airborne transport or avian-mediated dispersal, can reach almost every place in the world, including both inland and coastal sites. However, if these mechanisms were dominant, one would expect a higher degree of similarity among all sites. In addition, selection based on ionic composition does not appear to explain these patterns, as inland samples displayed similar ionic composition (Fig. 2) yet harbored highly dissimilar host and viral communities (Supplementary Fig. 1B). Therefore, while these pathways, as alternatives to marine currents, may contribute to viral dispersal, their role appears secondary to that of seawater-mediated transport.

Second, the analysis of an HSP sample collected from a small puddle on the rocky coast of Balearic Islands (Spain), named Coco 2 (CO2), of ephemeral existence and formed by the rapid evaporation and concentration of seawater, revealed that it shared 14 MAGs of extreme halophiles with other coastal sites [25], as well as viruses also present in geographically distinct samples (this study). With respect to its viruses, 13 vOTUs from CO2 were detected in other coastal hypersaline environments, and 54 CO2 vOTUs were tentatively assigned to halophilic hosts (mainly *Halorubrum* sp.) based on the presence of CRISPR protospacers. These findings provide compelling evidence that seawater not only harbors haloviruses, confirming and extending previous reports of halophiles in seawater [23], but also facilitates their dispersal across coastal sites. This highlights a previously underappreciated mechanism for the connectivity of hypersaline viral communities, emphasizing the role of seawater in the global distribution of haloviruses.

**Figure 6:**
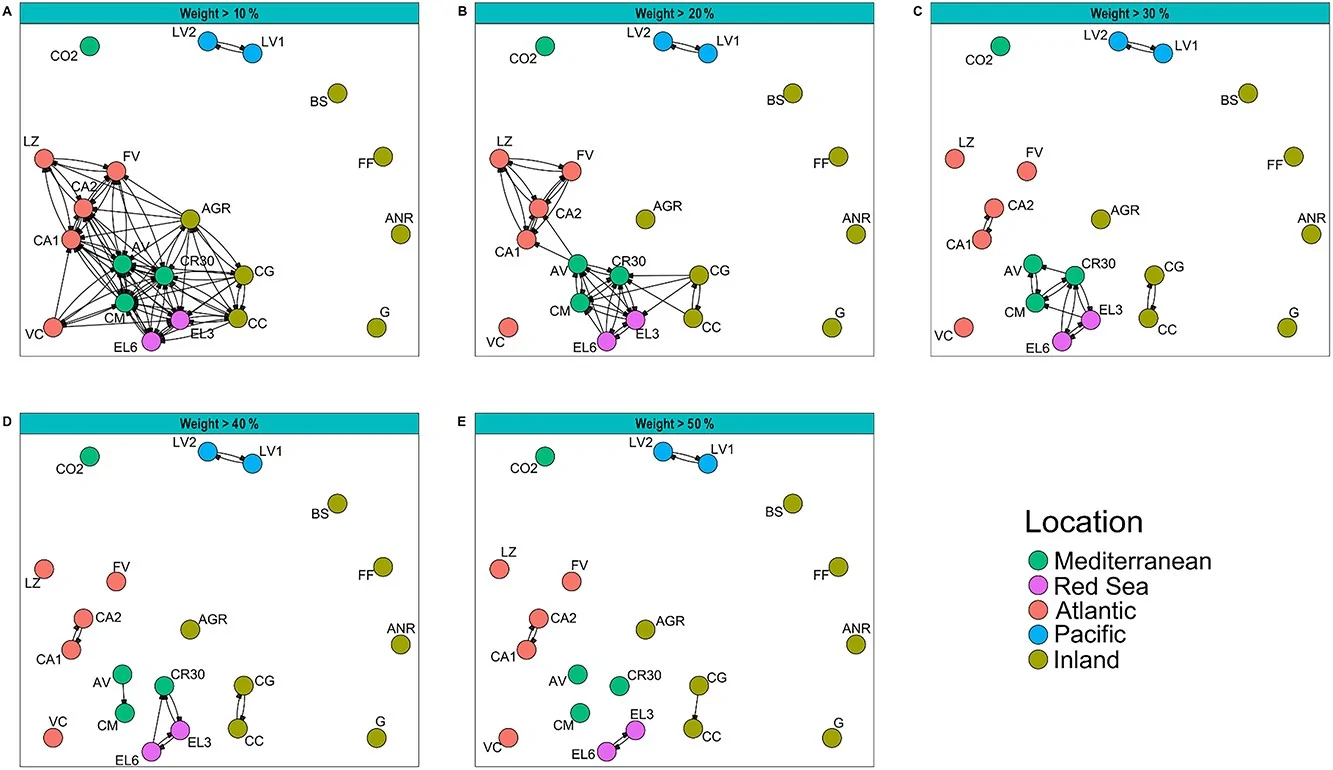
Connectivity of the viral community between sampling sites. In the networks, vertex represents sampling sites, whereas edges (arrows) indicate connected sites based on the percentage of the community shared (panel titles). A range of thresholds from 10% to 50% shared community were applied (see panels A–E). The direction of the arrow indicates the direction of the comparison. For example, in panel A, an arrow is displayed from sample LV1 to sample LV2 indicating that 10% of the total vOTUs present in LV1 are also detected in LV2. In addition, another arrow from LV2 to LV1 is also shown, indicating that the opposite is also true, that is, >10% of the vOTUs in LV2 are present in LV1. Note the high connectivity among coastal sites, whereas inland are more isolated (except CC and CG).

Third, three biogeographic patterns aligned well with the dynamics of marine currents in the Mediterranean and the Atlantic. Specifically, EL3/EL6 (Eilat, Israel) and CR30 (Santa Pola, Alicante, Spain) viromes shared around 40% of their vOTUs, whereas Eilat viromes shared lower percentages with those from the Balearic Islands (Spain) and with those located a few km away from CR30 (Fig. 6). Mediterranean Sea currents display a circular movement, where superficial seawater from the Atlantic Ocean enters the Mediterranean Sea through the Strait of Gibraltar, bordering the coast of Africa, and returning to the west by bordering the coast of Asia and Europe (Supplementary Fig. 4) (see [72–74] for more details). After entering the Strait of Gibraltar, surface seawater collides with the Alboran gyre and mixes with currents that flow south along the southeast coast of Spain, including Santa Pola, where CR30 is located [73, 74]. Then, as mentioned above, seawater borders the African coast, reaching the Suez Canal, which connects the Red Sea to the Mediterranean. At this point, haloviruses from CR30 could be transported to Eilat salterns by this water channel. On the other hand, the AV virome (Balearic Islands) shared >20% of its vOTUs with CA1/CA2 salterns (Canary Islands, Spain) (Fig. 6). Similarly, ten vOTUs detected in CO2 were also present in AV and samples from the Canary Islands, including CA2, FV, and LZ. However, none of the vOTUs detected in CO2 were present in the CR30 or Israel salterns. These findings suggest greater connectivity between the Balearic and Canary Islands than in other samples in our dataset. It is documented that the Mediterranean Outflow Water, which includes seawater from the Balearic Islands [75], exits the Mediterranean through the Strait of Gibraltar and interacts with the North Atlantic Gyre as it circulates toward the Canary Islands. This current, also called the Canary Current, might facilitate the dispersion of haloviruses from the Strait of Gibraltar to the Canary Islands, although not in the opposite direction. In addition, we observed that the inland sample AGR, despite being geographically closer to CR30 and the Balearic Islands than any other sample in our dataset, shared a low fraction (<20%) of the viral community with these two sites.

Collectively, these biogeographic patterns would not be expected if airborne or bird-mediated dispersal were the primary mechanisms of dispersal, as distance plays a greater role in such cases. In addition, experimental evidence has shown that some haloviruses remain intact at low salinity and regain infectivity as salinity increases [76, 77]. Such a property would allow them to structurally resist seawater during transport. Therefore, our results support the hypothesis that seawater could be an important vector for the dispersal of haloviruses among coastal hypersaline environments.

### Halovirus communities in inland environments exhibit a higher infection potential than those in coastal sites

**Figure 7:**
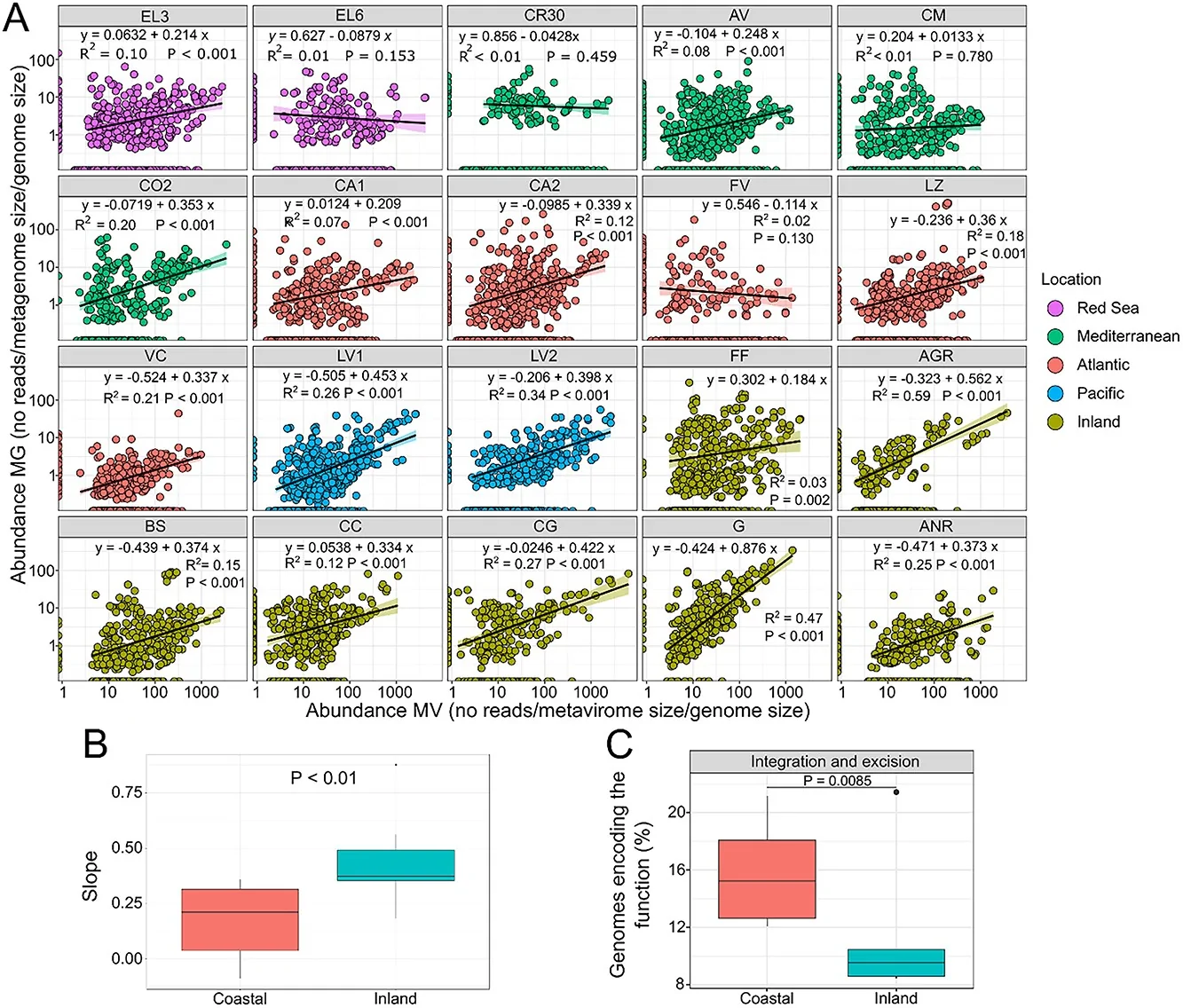
Distinct viral activity patterns between coastal and inland salterns. (A) Linear correlation of vOTUs relative abundance in the metagenome (y-axis) versus the relative abundance in the metavirome (x-axis). Each panel represents one sample and colored dots indicate the corresponding location (Red Sea: Violet; Mediterranean: Sea green; Atlantic: Salmon; Pacific: Sky Blue; and Inland: Olive green). Note the lack of correlation in most coastal samples (*P*-value >.05) as opposed to the strong correlation observed for inland salterns (*P*-value <.05). (B) Boxplot of the slopes observed in A for coastal and inland salterns, which showed statistically significant differences based on ANOVA. Each point in the boxplot represents the slope of one sample. (C) Boxplot showing the statistically significant enrichment (Wilcoxon test) of genomes encoding integration and excision genes in coastal samples. Data points in the boxplot represents the percentage of genomes encoding the corresponding gene in each sample. All vOTUs detected in the metagenome were considered. The same result was obtained when only the abundant (see methods for our definition of abundant) vOTUs detected in the metagenome were analysed, supporting the robustness of this observation.

Viral activity and life strategies in the GHV were inferred by comparing the relative abundances of vOTUs in metagenome-virome pairs from each sampling site (Fig. 7A). Those vOTUs detected exclusively in the virome (extracellular fraction), but not in the paired metagenome, were considered non-infective at the time of sampling. This pattern may result from several non-mutually exclusive scenarios: (i) the corresponding viral species was infecting too few hosts to be detected in the intracellular fraction; (ii) the virus had lost infectivity against the present hosts, while virions may persist in the environment due to high particle stability; or (iii) the virus was allochthonous. Conversely, vOTUs detected only in the metagenome (intracellular fraction) and absent from the paired virome were interpreted as (pseudo)lysogenic viruses that had not been induced at the time of sampling or shortly before. Finally, vOTUs detected in both extracellular and intracellular fractions were considered part of the active haloviral fraction, encompassing viral species actively produced and released through lytic replication, including induction of (pseudo)lysogens, or chronic infections, regardless of the number of infected cells, burst sizes, or environmental stability.

Most vOTUs of the GHV were detected exclusively in the extracellular viral fraction of each site (average per sample: 55.1% ± 20.1%), suggesting a limited intracellular representation of the virome-derived viral community at the time of sampling (Supplementary Fig. 5). Significant differences were observed between coastal and inland environments, with a higher proportion of inactive viruses in coastal salterns. This pattern may partially result from the transport of viruses from low- to high-salinity ponds during the seawater concentration process in salterns. However, because 89% of the vOTUs detected exclusively in viromes could not be confidently assigned to hosts, this explanation remains speculative. In contrast, no significant differences between coastal and inland sites were detected in the proportion of vOTUs found only in the cellular fraction (average per sample: 8.4% ± 9.4%) or in the proportion of presumably active viruses (average per sample: 36.2% ± 19.1%).

Focusing only on the active haloviral fraction, linear regressions were fitted to the central points (i.e. vOTUs detected in both extracellular and intracellular fractions; Fig. 7A) to assess if vOTUs’ intracellular relative abundance (y-axis) was explained by their extracellular relative abundance (x-axis). Our working hypothesis is that, assuming extracellular viral particles are predominantly derived from ongoing infections, a stronger linear correlation between extracellular and intracellular viral abundances may reflect a higher potential for viral infection and production in the system.

Statistically significant correlations (*P* <.05) were detected at 16 of the 20 sites analyzed. Moreover, coefficients of determination (R^2^) were, on average, higher in inland environments than in coastal ones (0.27 *versus* 0.12, respectively). Although R^2^ values were generally low, this pattern suggests that variation in intracellular viral abundance may be better explained by extracellular viral abundance in inland systems. In contrast, many coastal sites exhibited R^2^ values close to zero, suggesting that factors other than extracellular viral abundance may contribute to shaping intracellular viral levels. In addition, regression slope values, which describe the proportional relationship between intracellular and extracellular abundances, were also analyzed. While positive or negative slopes indicate a direct or inverse relationship between the two viral fractions, respectively, slope values close to zero reflect the absence of a linear relationship. As shown in Fig. 7B, slope values differed among sites and were significantly higher in inland environments (ANOVA, *P*-value <.05). This result was further supported by a statistically significantly higher Pearson correlation coefficient in inland *versus* coastal samples (Supplementary Fig. 6; *t-*test *P*-value .0169).

Taken together, the higher linear correlation in inland environments compared to coastal ones may indicate that inland systems exhibit higher viral infection and production, lower virion stability, or a combination of both. Conversely, coastal systems display weaker or non-significant correlations (Linear model, *P*-value >.05). One possible explanation is that coastal viromes may harbor a greater proportion of non-infectious viral particles within an “active” vOTU at the time of sampling. That is, a vOTU can be considered a species-like cluster containing several different intra-species units [78, 79] with varying infectivity. Therefore, a viral species classified as “active” could include, in the extracellular fraction, virions with high environmental stability lacking infective capacity that would nevertheless contribute to the relative abundance of that species.

We acknowledge that our interpretations have limitations. Specifically, these analyses describe relationships between intra- and extracellular viral relative abundances at a single time point and do not necessarily assume a linear correlation. Moreover, the observed patterns likely reflect both current and past infections, as extracellular viral particles may persist for varying periods. Viral decay rates are unlikely to be homogeneous across vOTUs and may influence the relative abundance in the extracellular compartment. Additional factors, such as host range or burst size, may also affect relative abundance patterns of active viruses. Because these parameters cannot be estimated from the available data, our interpretations cannot be conclusively validated. Nevertheless, functional annotation revealed a lower potential for integrative lysogeny in inland environments, as vOTUs from these sites encoded fewer integration and excision genes than those from coastal environments (Fig. 7C). In addition, viruses trapped in the diapirs that later fed inland systems upon dissolution by groundwater could act as a constant inoculum of infective phage particles that rapidly enter the lytic cycle, as observed in the underground saline waters of the Añana Ancient Salt Valley [43]. These observations provide independent support for the interpretation that inland systems have a higher potential for viral production than coastal systems.

Finally, viral OTUs associated with specific hosts allowed us to hypothesize the extent to which host identity or environment (coastal *versus* inland) might influence virus strategy. Viruses associated with *Haloquadratum*, *Salinibacter*, and *Halorubrum* species showed no correlation between viromes and metagenomes in coastal salterns, whereas a positive correlation was observed in the inland salterns of Romania (FF), Algeria (AGR), South Africa (BS), and Argentina (CC) (Supplementary Fig. 7A). These contrasting patterns suggest that the environment could modulate infection strategy. In contrast, viral microdiversity patterns, as measured by ANIr (see methods), appeared more host-dependent. Viral OTUs associated with *Hqr. walsbyi* tended to display higher microdiversity in the extracellular fraction than in the metagenomes, while *Halorubrum*-infecting viruses showed the opposite trend (Supplementary Fig. 7B-C). These results are consistent with the hypothesis that only a small fraction of viruses infecting *Haloquadratum* are active at any given time, while a diverse non-infective pool persists extracellularly [78], whereas *Halorubrum*-infecting viruses might generate diversity during infection with few particles persisting outside the cells. These observations, however, do not account for the genomic heterogeneity of the host populations considered. Indeed, generalist hosts present in coastal and inland systems comprise multiple strains or genomovars [25] which most likely present distinct susceptibilities to viral infection, partly explaining the patterns observed. These interpretations should therefore be kept as working hypotheses requiring further validation.

## Concluding remarks

This study presents the GHV as the most comprehensive characterization of halophilic viral diversity to date. Our results indicate that haloviral communities are shaped by a combination of environmental selection and dispersal dynamics, revealing fundamental differences between coastal and inland systems. Coastal hypersaline sites may be interconnected via seawater-mediated transport, allowing haloviruses to disperse across vast distances and create a more cohesive viral network. In contrast, inland aquatic hypersaline environments are dominated by highly endemic viral assemblages, reflecting local adaptation and higher viral specialization. Moreover, inland viral communities exhibit higher infection potential and reduced integrative lysogeny, suggesting a more dynamic viral production, whereas coastal systems are characterized by a greater proportion of non-infectious vOTUs.

Together, these findings redefine our understanding of viral biogeography in hypersaline ecosystems, illustrating how environmental connectivity, host specificity, and ecological strategy shape the haloviral landscape across contrasting environments (coastal *versus* inland). The observed patterns offer valuable insights into viral dynamics and highlight ecological trends consistent with recent studies (e.g. [70, 78]), which emphasize that reliance on virome analyses alone may lead to misinterpretations of viral community ecology and evolution. Therefore, integrating abundance-based analyses with functional indicators is essential for accurately inferring viral activity and infection strategies across diverse ecosystems.

## Supplementary Material

Supplementary_material_ycag244

## Data Availability

Metagenomic data supporting this article are available in the European Nucleotide Archive (ENA) at https://www.ebi.ac.uk/ena/browser/home under BioProject accession number PRJEB45291. Accession numbers for each individual virome are listed in Table 1. The GHV is publicly available in Zenodo at doi:10.5281/zenodo.21450953
