# Supplementary material for: Dispersal pathways and infection strategies within the Global Halophilic Virome
Source: ISME Commun. 2026 Aug 25;6(1):ycag244. doi: 10.1093/ismeco/ycag244 (PMC13600414; doi:10.1093/ismeco/ycag244)
Supplement: Supplementary file 1 [file supplementary_material_ycag244.zip › Aldeguer-Riquelme_et_al_2026_-_New_Suppl_Figures_ycag244.docx]

**Dispersal pathways and infection strategies within the Global Halophilic Virome**

Borja Aldeguer-Riquelme^1,2,3^, Cristina López^1^, Eva Mayol^1,4^, María Dolores Ramos-Barbero^1,5^, Valentín Gangloff^1^, Tomeu Viver^6^, Ana S. Ramírez^7^, Souad Amiour^8^, Aharon Oren^9^, Stephanus N. Venter^10^, María E. Llames^11^, Bernardo González^12^, Horia L. Banciu^13,14^, Catherine Lizama^15^, Ramon Rossello-Mora^6^, Fernando Santos^1,16*^, Josefa Antón^1,16,17^

^1^ Department of Physiology, Genetics, and Microbiology, University of Alicante, San Vicente del Raspeig 03690, Alicante, Spain

^2^ School of Civil & Environmental Engineering and School of Biological Sciences, Georgia Institute of Technology, Atlanta, Georgia, USA

^3^ Department of Biomedicine and Dentistry, Faculty of Biomedical Sciences and Sports, Universidad Europea de Andalucía, Calle Eolo 10, 29010-Málaga, Spain

^4^ HUB Ambiental UPLA, Universidad de Playa Ancha, Valparaíso, Chile

^5^ Departament de Genètica, Microbiologia i Estadística, Universitat de Barcelona, Diagonal 643. Annex. Floor 0, E-08028 Barcelona, Spain

^6^ Marine Microbiology Group, Department of Animal and Microbial Biodiversity, Mediterranean Institute for Advanced Studies (IMEDEA, UIB-CSIC), Esporles, Spain

^7^ Unidad de Epidemiología y Medicina Preventiva, IUSA, Facultad de Veterinaria, Universidad de Las Palmas de Gran Canaria, C/Trasmontaña s/n, Arucas, 35413, Canary Islands, Spain

^8^ Laboratory of Bioengineering, National Higher School of Biotechnology, Constantine, Algeria

^9^ The Institute of Life Sciences, The Hebrew University of Jerusalem, The Edmond J. Safra Campus, 9190401 Jerusalem, Israel

^10^ Department of Biochemistry, Genetics and Microbiology, and Forestry and Agricultural Biotechnology Institute (FABI), University of Pretoria, Pretoria, South Africa

^11^ Laboratorio de Ecología Microbiana Ambiental, Instituto Tecnológico de Chascomús (CONICET-UNSAM), Escuela de Bio y Nanotecnologías (UNSAM), Buenos Aires, Argentina

^12^ Facultad de Ingeniería y Ciencias, Universidad Adolfo Ibáñez–Center for Applied Ecology and Sustainability, Santiago de Chile, Chile

^13^ Department of Molecular Biology and Biotechnology, Faculty of Biology and Geology, Babeș-Bolyai University, Cluj‑Napoca, Romania

^14^ Emil G. Racoviță Institute, Babeș-Bolyai University, 5-7 Clinicilor Street, 400006, Cluj-Napoca, Romania

^15^ Departamento de Tecnología Médica, Facultad de Ciencias de la Salud, Universidad de Antofagasta, Antofagasta 1240000, Chile

^16^ Applied Biomedicine Group (Alicante Institute for Health and Biomedical Researc h, ISABIAL), Alicante 03080, Alicante, Spain

^17^ Multidisciplinary Institute of Environmental Studies Ramon Margalef, Alicante 03690, Spain

*Corresponding author: Fernando Santos ([fernando.santos@ua.es](mailto:fernando.santos@ua.es))

**Supplementary Figure 1.** Nonmetric multidimensional scaling (NMDS) based on the MASH distance values calculated from the raw reads of viromes from a range of environments, including freshwater, seawater, hypersaline water and soils (A) and for the twenty halophilic viromes analyzed in this study (B). In panel A, colors indicate the environment the sample was collected from. Note, the clustering of samples belonging to the same environment. In panel B, colors indicate the location whereas shapes indicate the type of sample (coastal vs inland). Note the dispersion of inland samples compared to the more homogeneous clustering of coastal samples.

**Supplementary Figure 2.** Differences in microdiversity among vOTUs detected in AV and CR30 that were assembled from either the same sample or a different sample. Microdiversity was estimated as the average nucleotide identity of reads mapped to each vOTU (ANIr) within each metavirome (AV or CR30). Each dot represents the ANIr value (y-axis) of a vOTU assembled from the same or different sample (x-axis). Only vOTUs with sequencing depth values above 10X were included in the analysis. Statistically significant differences were observed between the two groups in both metaviromes (t-test, p-value < 0.001), with vOTUs assembled from different samples exhibiting lower ANIr values. These results support the hypothesis that high microdiversity (i.e., lower ANIr) hampers the assembly of viral contigs.

**Supplementary Figure 3.** Nonmetric multidimensional scaling (NMDS) based on the distribution and abundance of the 10,705 vOTUs included in the Global Halovirus Database (GHV) in the twenty metaviromes analyzed in this study. Colors indicate the geographic location of the sample. Note the similarities with the NMDS plot obtained with MASH distances calculated from raw reads (Supplementary Figure 1B).

**Supplementary Figure 4.** Map of the Mediterranean Sea displaying marine currents (arrows) along the geographical position of the hypersaline systems analysed in this study (dots). Surface and deep currents are indicated with blue and red colours, respectively. Coloured dots represent different geographical locations (Atlantic, Mediterranean, Red Sea or Inland). CR30, EL3 and EL6 shared over 40% of their viral communities, suggesting a connection between them (see Figure S5). CR30 and Israel salterns (EL3 and EL6), may be linked by the surface current flowing west to east along the African coast and then through the Suez Canal. Hypersaline system in the Balearic Islands (AV) shared over 20% of its vOTUs with samples in the Canary Islands (see Figure S5), possibly due to a connection via the Mediterranean Outflow Water and the Canary Current. Marine currents displayed in this illustration are based on previous studies by Pinardi and Masetti (2000)[43], Marta Pascual *et al* (2017)[44] and Auriane Virgili (2018)[45].

**Supplementary Figure 5.** Venn diagrams depict the number and percentage (in parentheses) of vOTUs that are exclusive to or shared between the metaviromes (MV) and metagenomes (MG) for each sample. The colors represent the relative contribution of each fraction (exclusive to metavirome, shared, or exclusive to metagenome), ranging from low (dark blue) to high (light blue).

**Supplementary Figure 6.** Boxplot showing the Pearson correlation coefficients (relative abundance in virome *vs* relative abundance in metagenome) in coastal and inland samples.

**Supplementary Figure 7.** Host-specific viral activity patterns. A) Lineal correlation of vOTUs relative abundance in the metagenome (y-axis) versus the relative abundance in the metavirome (x-axis) of vOTUs associated to *Haloquadratum walsbyi*, *Salinibacter ruber* and *Halorubrum* spp. Each panel represents one sample and colored dots indicate the corresponding host. Note that host-specific correlations remain similar for each sample, indicating a larger importance of the environment to determine the viral activity strategy rather than the host. B) Boxplots of intraspecies diversity, measured as the average identity of mapped reads (ANIr, y-axis), of vOTUs associated to each host (x-axis). Colors indicate intracellular (metagenome) or extracellular (virome) source. Only vOTUs detected in both the metagenome and metavirome and with a minimum sequencing depth of 10 were considered. Note the higher intraspecies diversity of vOTUs associated to *Hqr. walsbyi* in the extracellular compared to the intracellular fraction whereas *Halorubrum* vOTUs showed the opposite pattern, that is, higher intraspecies diversity in the intracellular fraction. Only vOTUs detected in both fractions were considered. C) Number of vOTUs per host showing higher ANIr in the intracellular or extracellular fractions. In addition to the filters applied in B, only vOTUs displaying ANIr differences larger than 0.1 were considered.
